# Approaching Cancer Evolution from Different Angles

**DOI:** 10.1016/j.isci.2020.101661

**Published:** 2020-10-22

**Authors:** Francesca D. Ciccarelli, James DeGregori

**Affiliations:** 1 Cancer Systems Biology Laboratory, the Francis Crick Institute, London, NW1 1AT, UK; 2 School of Cancer and Pharmaceutical Sciences, King's College London, London SE11UL, UK; 3 Department of Biochemistry and Molecular Genetics, University of Colorado Anschutz Medical Campus, Aurora, CO 80045, U S A

## Abstract

Dr Francesca Ciccarelli (The Francis Crick Institute, UK) and Dr James De Gregori (University of Colorado, USA) interview 3 top scientists in clinical (Dr Charles Swanton, The Francis Crick Institute, UK), molecular (Dr Kornelia Polyak, Dana-Farber Cancer Institute, USA), and evolutionary cancer research (Dr Carlo Maley, Arizona State University, USA) to discuss the current status of knowledge, the challenges, and the opportunities to move the field forward.

**Figure:**
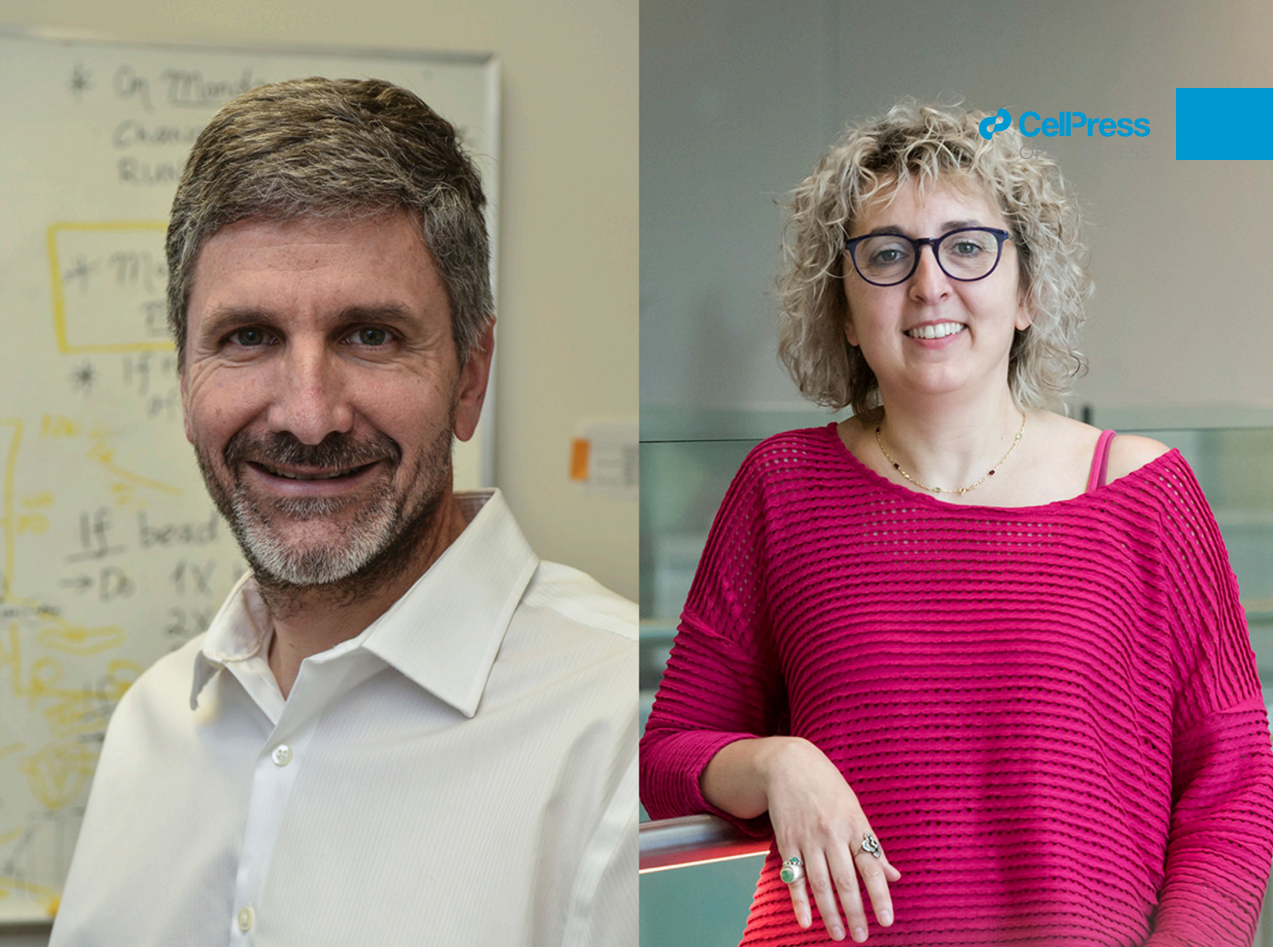

## Main Text

### What Recent Results in Cancer Evolutionary Biology Have Gotten You the Most Excited and Why?

#### Carlo Maley

I am struck by the result from Martincorena et al.’s study (Martincorena, 2018b) addressing a long standing prediction – that the ratio of beneficial vs. deleterious mutations in somatic evolution should be quite different from organismal evolution (Leroi et al., 2003; Merlo et al., 2006). In most cases, organisms have been optimized by natural selection such that most mutations that have a phenotypic effect (non-neutral mutations), will tend to make things worse. There are typically very few beneficial mutations possible for most organisms, and many deleterious ones (Böndel et al., 2019; Eyre-Walker and Keightley, 2007). However, natural selection has not optimized the fitness of somatic cells. They do not proliferate and survive as best as possible in our bodies. Quite the contrary, their proliferation is tightly regulated and they often die at the first hint of anything going wrong. That is because natural selection has optimized them to cooperate in the fitness of the organism (Aktipis, 2020). So, there should be many more mutations in a somatic cell that increase its cellular fitness, compared to mutations that increase the organismal fitness. They could even be more frequent than mutations that are deleterious for the cell. If so, then a mutator mutation, that increased the mutation rate of a somatic cell, would be positively selected for, as it produced more adaptive mutations than deleterious mutations. This is counterintuitive for evolutionary biologists, but Martincorena's study suggests that it is true. They found little evidence of negative selection, weeding out deleterious mutations, except in a few essential genes. But they found lots of evidence of positive selection, enriching mutations that increased the fitness of somatic cells.

“Natural selection has not optimized the fitness of somatic cells”

“Many of our lifestyle factors that influence risk and survival (e.g., diet, exercise) at least in part do so via the immune system.”

“Metronomic therapies and cycling or alternating therapies with drug holidays are new approaches being tested in investigator-initiated trials.”

#### Kornelia Polyak

I really like the studies analyzing the co-evolution of cancer cells and the immune environment. Like studies showing particular cancer clones being eliminated due to adaptive immune response based on T cell receptors (TCRs) or showing that there is an optimal degree of subclonal neoantigen heterogeneity – too much or too little are not good. To me these are proving evolution happening in real time.

#### Charles Swanton

There are two recent results that I find particularly exciting.The first concerns the ability to monitor branched evolution in real time from ctDNA in blood and track minimal residual disease after surgery by monitoring the emergence of clonal and branched mutations in ctDNA. This opportunity offers the ability to intervene when the burden of disease is low following surgery with curative intent to escalate or guide the judicious use of adjuvant therapy targeted toward those patients most likely to relapse with metastatic disease. This would allow trials to be structured in such a way as to test the evolutionary principle of rendering the emerging tumor extinct when the population size is at its lowest, and when pre-existing drug resistance mechanisms are less likely to be present.

The second recent development that I find exciting is extrachromosomal DNA (ECDNA). The extent of ECDNA in tumor is becoming apparent from work from Paul Mischel and colleagues. This presents a formidable challenge to our understanding of evolutionary biology, since these fragments lack centromeres and cannot bind the mitotic spindle, and hence their segregation into daughter cells at mitosis is unpredictable. Added to which they reintegrate intrachromosomally into homogeneously staining regions and can reappear again as ECDNAs following drug exposure. Understanding the mechanistic basis to their formation and re-integration is going to be critical for cancers of unmet need such as glioma/glioblastomas, lung and esophageal cancers amongst many others.

#### Kornelia Polyak

What I find the most surprising is that these clones can expand and persist despite the fact that several of these mutations that are detected (e.g., PIK3CA hotspots) are predicted neoantigens. Why does the immune system not recognize these clones as abnormal? Why are they not eliminated? Or the fact that we can detect these is already an indication of a permissive immune suppressive environment possibly due to age and lifestyle factors?

#### Carlo Maley

The first big observation, although not a big surprise, is that normal tissue often contains many oncogenic mutations. We knew the skin often has TP53 mutations, but the studies from Martincorena's lab and others showed this was true for many cancer genes (Lee-Six et al., 2019; Martincorena et al., 2015; Martincorena, et al., 2018a; Yokoyama et al., 2019). The big surprise was that for some oncogenes, like NOTCH1, mutations are more common in the normal tissue than in the cancers that grow out of that tissue! These genes had been identified as cancer genes due to their high frequency of mutations in skin and esophageal squamous cell carcinomas. But we were missing the appropriate control condition – their frequency of mutation in the normal tissue. There is no doubt that mutations in NOTCH1 drive clonal expansions, but the implications of these results are that those expansions are cancer protective. This highlights an important point that Mary Kuhner made – just because a mutation increases the fitness of a somatic cell and causes a clonal expansion does not necessarily make it a driver of carcinogenesis (Kuhner et al., 2016). This may also be the case with CDKN2A alterations in Barrett's esophagus. We have hypothesized that clonal expansions of CDKN2A mutants are not carcinogenic in and of themselves, but it is important which mutations hitch a ride on that expansion. If the CDKN2A mutant clone also contains a mutation in TP53, the expansion of the TP53 mutant clone can dramatically increase the risk of progression to cancer (Maley et al., 2004). However, if the expanding clone with a CDKN2A mutation is TP53 wildtype, then TP53 has missed an important chance to hitch a ride, and may be less likely to be able to expand in the future. In that case, a CDKN2A clonal expansion might even be protective from cancer. In our analyses, a clonal expansion of CDKN2A mutant clone was not predictive of progression once we controlled for the presence of lesions in TP53 (Maley et al., 2004).

Recent studies have shed a lot of new light on mutational and somatic evolutionary processes in normal tissues. What surprised you from these studies, and how has our understanding of aging and tumor initiation been altered?

#### Charles

Somatic mutations do not appear to distinguish tumor from normal cells well, but somatic copy number aberrations may be somewhat better. This work illustrates the need to understand why some cells transform to a fully fledged malignancy and others do not. Is this due to the cell of origin in which these mutations occur? Is this due to the order in which mutations occur? Or are we thinking about cancer evolution wrongly and could an aging soma be required to permit clonal expansions with the right somatic mutational background?

#### Kornelia Polyak

A lot. In my view tumor progression is limited by the immune system, thus, immune responses are the most effective natural defense against clinically detectable cancer. We see that the primary tumor can modify even the systemic immune environment and make it more permissible to disease progression. We also see that host “immune history” (e.g., childhood infectious diseases, antibiotic use) influences both cancer incidence and progression. Many of our lifestyle factors that influence risk and survival (e.g., diet, exercise) at least in part do so via the immune system. Regarding this, we should study more people who do not get cancer despite risk factors. What's special about them?

#### Carlo Maley

There is clearly a complex interaction between the immune system and the evolution of neoplasms. There is evidence for immunoediting - for the immune system recognizing some non-synonymous mutations in somatic cells and removing those cells (Schreiber et al., 2011). However, there are also multiple mechanisms of immune-escape that can evolve in neoplasms. There are also a lot of other sources of variation in a neoplasm's microenvironment including fibroblasts, endothelial cells, extracellular matrix, etc. These must impose differential selective pressures on the neoplastic cells in the different microenvironments. In addition, many of those cells in the microenvironment can be recruited, repelled or reprogrammed by the neoplastic cells, in a somatic version of niche construction. Thus, neoplastic cells can change the selective pressures on themselves. Currently, we know very little about to what extent the heterogeneity of the microenvironment generates and selects for heterogeneity of clones in a neoplasm. However, this is important, not only because the microenvironment can sometimes control the phenotype of the neoplastic cells (Bissell and Hines, 2011) but also because the heterogeneity within a neoplasm provides evolutionary resilience to any changes in its environment, including our therapeutic interventions.

#### Charles Swanton

In lung cancer, we have some evidence for this. Firstly, we see HLA LOH in 40% of untreated patients, heavily enriched in smokers or ex-smokers (McGranahan et al., 2017). We see evidence for antigen presentation machinery disruption in immune hot tumors and clonal neoantigen deletions in immune cold tumors (Rosenthal et al., 2019). We also see that patients with tumors with a high clonal mutational burden benefit more from cancer immunotherapy and have better clinical outcome after surgery with curative intent in the absence of immune therapy, supporting the possibility that clonal neoantigens may allow optimal immune surveillance(McGranahan et al., 2016; Rosenthal et al., 2019).

How much do you think the immune system is shaping the mutational landscapes in our bodies (from mutation occurrence to full cancer development) and vice-versa?

What are the biggest unanswered evolutionary questions in cancer biology right now?

#### Kornelia Polyak

The following are, in my opinion, some of the most urgent questions we should be answering.

To what degree tumor evolutionary paths are predetermined at early stages of tumor initiation and to what degree this is dependent on the host or the tumor itself? How can we effectively modulate intratumor heterogeneity and thus, decrease the risk of disease progression and the emergence of resistance?

How can we predict what is the best possible combination therapy to use for an individual patient and when to apply it?

Why are some tumors immune cold and do not respond to immunotherapies?

How can we control the evolution of therapeutic resistance to prevent patient death due to cancer? Most cancer deaths are due to therapeutic resistance (Wang et al., 2019). Yet, it seems like most cancer therapy research is based on finding a new drug target, ignoring the fact that every cancer drug that has been invented selects for resistance. We need to face this fact head on and figure out how to prevent or control therapeutic resistance. The pest managers are about 30 years ahead of us, and I think we have a lot to learn from them, beyond just adaptive therapy (West et al., 2020).

What mechanisms has evolution discovered for suppressing cancer (in non-human organisms)? The pest managers are one source of inspiration, but mother nature is another. Evolution has discovered ways of suppressing cancer many times over. Every time a large and/or long lived organism has evolved, it has had to evolve mechanisms to suppress cancer long enough to successfully reproduce (Caulin and Maley, 2011; Nunney et al., 2015; Roche et al., 2012; Tollis et al., 2017). The evolution of long life span and large body size has occurred independently many times. This is why we have started to study elephants (Abegglen et al., 2015; Sulak et al., 2016), whales (Marc Tollis et al., 2019), and other surprisingly cancer-resistant organisms. We hope to translate any discoveries into new methods for cancer prevention for humans.

#### Charles Swanton

We still have very little understanding of the metastatic disease process in patients. We currently lack large scale well annotated pan-cancer longitudinal cohorts from diagnosis through to cure or death. We are attempting to address this in non-small-cell lung carcinoma (NSCLC) and renal cancer through studies such as TRACERx. Roel Verhaak is leading the Glass consortium that is looking at this problem in glioma. However, we need to address this in other cancers too. Ultimately, we must attempt to access metastatic tissue in a broader manner. One way, this will be possible is through autopsy analyses such as the PEACE program in the UK led by Mariam Jamal-Hanjani and the Donum study in Italy led by Alberto Bardelli, as well as pancreas studies led by Christine Iacobuzio Donahue in the USA. These are challenging and require extensive collaboration and funding support.

#### Kornelia Polyak

I think there is some progress in this area, but it is still in its infancy. For example, there are clinical trials showing that intermittent dosing may be better for targeted therapies than continuous treatment (e.g., BRAF mutant melanoma and castration-resistant prostate cancer). There are also some trials based on rational combinations.

My view is that we should assume that there is a resistant clone from the start, since especially for large tumors the probability of missing a minor subclone based on a single biopsy is very high. Thus, I would like to see more focus on developing rational combinations that are non-toxic and apply these early during treatment. Our current clinical management of most solid tumors is – treat until they are resistant and then switch to another drug – many times each one of these are single agents. There are multiple problems with this approach. First, we know that there will be resistance to single agent treatment – the larger the tumor burden, the more likely this occurs and occurs fast. Second, even non-effective treatments change the tumor (and the body) in a way that may select for “fitter” cancer cells making subsequent treatment more difficult. Third, new drugs are commonly tested as single agents in metastatic treatment resistant disease and then we ask a single agent to have efficacy to shrink the tumor. I fear that many drugs that could work at earlier stages or be effective as combinations, but not as single agents, are thrown away due to this process.

Is the evolutionary view of cancer already altering the way we treat patients and what should be done more or differently in this direction? For example, should the development of personalized approaches to patient treatment still be a priority, considering the rapid onset of resistant subclones?

#### Carlo Maley

I think we need to expand our concept of what “personalized” means in cancer therapy. Typically, it has meant that we sequence a tumor, look for mutations that we can target with a drug, and apply that drug. If that mutation is not clonal, if it is not in 100% of all the cancer cells, then the inevitable result is relapse due to the cells that don't have that mutation. Even if the mutation is clonal, there is very often at least one clone (often many) that has an additional mutation that makes it resistant to the drug of choice. Those resistance mutations ought to be screened for, but they are so rare, they may be near impossible to detect. I prefer the personalized medicine that emerged from the adaptive therapy clinical trial (Zhang et al., 2017). They adapted the drug dosing to the individual kinetics of each patient's tumor. Some tumors shrank rapidly, but also regrew rapidly. Some took a long time to shrink but also a long time to grow back. No fixed protocol could effectively treat the diversity of tumors in the clinic. The adaptive therapy protocol automatically personalized the treatment for each patient. This reveals the importance of rapid, inexpensive and frequent assays to monitor cancers during treatment. I'm hopeful that, in the near future, cell free DNA in the blood (Bos et al., 2020; Cervena et al., 2019; Jamal-Hanjani et al., 2016; Stastny et al., 2020) might be used to monitor how the clones are evolving in our patients. Cancers evolve in response to our interventions. Our interventions must evolve in response to the cancers.

#### Charles Swanton

Changing the drug development paradigm is not straight forward. Most phase II studies are conducted based on phase I trials establishing the maximum tolerated dose of drug given continuously or at weekly/monthly intervals with breaks in between according to tolerance. Treatment is continued, aimed at maximizing tumor response. However, there is a compelling argument pioneered by colleagues at Moffitt cancer center that cancer drug development should learn lessons from ecology and pest control (West et al., 2020). Put simply, therapies in advanced disease allow resistant subclones to emerge that the clinician is now much less capable of managing. Therefore, why not try to compete sensitive subclones with resistant ones rather than destroying the sensitive subclones allowing the resistant ones to emerge in a competition-free environment? Metronomic therapies and cycling or alternating therapies with drug holidays are new approaches being tested in investigator-initiated trials. However, these studies often require reliable markers or surrogates of cancer clonal evolution in order to detect resistant subclones as they emerge so that drugs can be alternated, changed or temporarily stopped. Our tools to measure the evolution of such subclones are still in their infancy. Circulating tumor DNA shows much promise in this regard. Minimal residual disease clinical trials are in progress or getting underway to test the paradigm that treatment escalation after surgery in the absence of disease on a CT scan can improve the chances of cures in patients with emergent ctDNA in blood that forecasts a high risk of future recurrence.

Finally, the immune system represents a finely tuned system to target the inexorable diversity present as cancers evolve and limit drug resistance. Here I should declare a conflict of interest as a co-founder of Achilles Therapeutics with Sergio Quezada and Karl Peggs. We recognized the importance of clonal neoantigens in 2016 and now efforts are on-going in the UK to test the ability of adoptive T cell therapy to target such clonal or truncal neoantigens present in every tumor cell, to limit the acquisition of drug resistance.
